# Micro-shear bond strength of different surface treatments on a polymer infiltrated ceramic network

**DOI:** 10.12688/f1000research.122108.1

**Published:** 2022-07-18

**Authors:** Dian Agustin Wahjuningrum, Calvo Ramírez Juan Norberto, Méndez Mendieta Luisa Fernanda, Amanda Andika Sari, Ajinkya M. Pawar, Alberto Carlos Cruz González

**Affiliations:** 1 Department of Conservative Dentistry, Faculty of Dental Medicine, Universitas Airlangga, Surabaya, Indonesia; 2 Oral Health Department, Faculty of Dentistry, Universidad Nacional de Colombia, Bogotá, Colombia; 3 Department of Conservative Dentistry and Endodontics, Nair Hospital Dental College, Mumbai 400008, Maharashtra, India

**Keywords:** Micro shear bond strength, polymer infiltrated ceramic network (PICN)

## Abstract

**Background:** Polymer infiltrated ceramic networks, or hybrid ceramics, are a combination of infiltrating polymerizable organic monomers into a pre-sintered porous ceramic matrix. In addition to having good mechanical properties, the polymer infiltrated ceramic network must comply with the possibility of adequate bonding to the resinous cement. The surface conditioning of this hybrid material must be carefully considered due to its organic composition and ceramic network. The purpose of this research is to evaluate the effect of hydrofluoric acid and a self-etching ceramic primer, under two different application times, on the bond strength of a polymer infiltrated ceramic network.

**Methods:** Blocks of a polymer infiltrated ceramic network were cut to obtain sheets, and these were randomized into five groups. For the group termed AAS, airborne-particle abrasion with Al _2_O _3_ (aluminum oxide) of 50µm was used. For groups HF2 and HF6, hydrofluoric acid was used for 20 and 60 seconds respectively, and for the groups MB2 and MB6, a self-etch ceramic primer was applied for 20 and 60 seconds respectively. A silane was applied to the groups AAS, HF2, and HF6 after the treatment. After 24-hour storage in distilled water, a micro-shear bond strength test was performed using a universal mechanical testing machine. All samples were evaluated in a stereomicroscope at 40x and 50x to determine the type of failure.

**Results:** The highest and lowest values of bond strength were reported by groups MB6 and AAS, respectively. Groups HF2, HF6, MB6, and MB2 did not report statistically significant differences. The predominant failure pattern was a mixed failure.

**Conclusions:** With the limitations of the present investigation, the treatments of self-etching ceramic primer and hydrofluoric acid followed by silane were reported to be statistically equal at 20 and 60 seconds.

## Editorial note

Editorial Note (26 ^th^ July 2023): The F1000 Editorial Team is currently substantiating that this research was presented at an IIARP conference and should be included in the IIARP Gateway. The Editorial Team is currently requesting further information from the authors regarding this. This Editorial Note will be updated as new information is provided.

## Introduction

PICN (polymer infiltrated ceramic network), or hybrid ceramics, were introduced in dentistry as a restorative material, which, by infiltrating polymerizable organic monomers into a pre-sintered porous ceramic matrix, promises to obtain mechanical properties like dental enamel and a density equivalent to that of dentine. ^1^ Hybrid ceramic offers a combined resistance to different failure modes, where the ceramic matrix confers resistance to wear and deformation, while the polymer provides some plastic deformation and reduces toughness or brittleness. ^2^^,^^3^ The PICN has a composition of 86%wt of feldspathic ceramic and 14%wt of infiltrate of dimethacrylates (UDMA and TEGDMA, which are urethane-dimethacrylate and triethylene glycol dimethacrylate, respectively). ^4^ It presents mechanical properties comparable to CAD-CAM (computer-aided design/computer-aided manufacturing) polymers with nano-ceramic particles, lower than lithium disilicate, but superior to CAD-CAM feldspathic ceramics. ^2^^–^^4^ Hybrid ceramics, in addition to having good mechanical properties, must comply with the possibility of adequate bonding to the resinous cement. ^5^

Adhesive cementation increases the surface energy and the retention of the restoration reinforces its structural strength and maintains marginal integrity. ^6^ Due to its high ceramic content, this type of material requires surface conditioning prior to resinous cement to ensure an adequate bond quality. ^7^ Etching with 5% hydrofluoric acid for 60 seconds is the PICN manufacturer's recommended surface conditioning. ^5^ However, other treatments such as hydrofluoric acid at 9% for 90 seconds, silanes, airborne-particle abrasion with aluminum oxide (30-50 μm particles) and tribochemical silica (CoJet System - 30 μm particles) have been reported. ^8^^,^^9^ The association of hydrofluoric acid or air abrasion (aluminum oxide) with a silane agent can achieve considerable values of bond strength, due to the structure with a high content of feldspathic ceramic present in this material (>80%wt), compared to other CAD-CAM materials with polymeric matrix and dispersed ceramic particles, with lower values of bond strength using this same combination of surface treatments. ^10^ However, given the risk of excessive dissolution of the vitreous matrix due to the attack of hydrofluoric acid, an etching agent and silane have been proposed in a single step, known commercially as Monobond Etch & Prime. ^11^ With a chemical in its composition less aggressive than hydrofluoric acid, tetrabutylammonium dihydrogen trifluoride, this self-etching ceramic primer has reported considerable values of bond strength in lithium disilicate and feldspathic ceramics. ^12^

The objective of this study was to evaluate the effect of hydrofluoric acid and a self-etching ceramic primer, under two different application times, on the bond strength of a polymer infiltrated ceramic network. The null hypothesis was that the mean values of bond strength of the groups treated with Monobond Etch & Prime would be equal to the groups treated with hydrofluoric acid and silane.

## Methods

### Ethics statement

We obtained ethical approval for this study from Faculty of Dentistry, Universidad Nacional de Colombia (approval number B.CIEFO-094-19). No patients or biological material obtained from patients were involved in this investigation.

### Research design

In the present study, 15 sheets (8 mm wide and 10 mm high) were made from three VITA ENAMIC EM14 blocks (VITA Zahnfabrik, Bad Sackingen, Germany) by means of cuts every 2 mm using a precision diamond disc with constant cooling with distilled water (Isomet, Buehler Illinois, USA). These sheets were included in cylinders of self-curing acrylic resin (Veracril, New Stetic, Guarne, Antioquia, Colombia), 10 mm high, leaving an exposed face of the hybrid ceramic to receive surface treatment. The exposed surfaces were sanded with #600, #800, #1000, and #1200 grain size silicon carbide abrasive paper for approximately 1 minute under manual pressure. The samples were washed in distilled water with ultrasound for 15 minutes.

### Experimental procedure

The sheets were randomized into five groups, with three sheets per group (n = 15) according to the surface treatments to be received. The group termed AAS had airborne-particle abrasion with Al _2_O _3_ of 50 μm applied for 10 seconds with a pressure of 1 bar, it was then washed with an air-water syringe for 20 seconds followed by drying with pressurized air for 10 seconds. A silane (Monobond N, Ivoclar Vivadent, Schaan, Liechtenstein) was applied with a microbrush rubbing for five seconds and leaving it to act for 60 seconds. Group MB2 used a self-etch primer (Monobond Etch & Prime, Ivoclar Vivadent, Schaan, Liechtenstein) which was applied for 20 seconds and then washed with water from an air-water syringe and dried with pressurized air for 10 seconds. Group MB6: like group MB2, but with the only difference that the primer was left to act for 60 seconds. Group HF2 used 9.6% hydrofluoric acid (Maquira Paraná, Brazil) which was applied for 20 seconds, it was then washed with an air-water syringe for 20 seconds, and dried with pressurized air, silane (Monobond N, Ivoclar Vivadent, Schaan, Liechtenstein) was applied with a microbrush rubbing for five seconds and it was left to act for 60 seconds, then air was gently applied for 10 seconds. Group HF6 was similar to group HF2, but with the difference that the etching with hydrofluoric acid was carried out for 60 seconds. The hydrofluoric acid residues were neutralized in a supersaturated solution of calcium carbonate.

On the conditioned surfaces, 0.75 mm internal diameter and 0.8 mm high medical grade silicone tubes were positioned and carefully filled with dual resinous cement (LuxaCore Z, DMG, Hamburg, Germany), finally, each cylinder was cured with the high setting for 20 seconds with a LED lamp (Bluephase C8, Ivoclar Vivadent, Schaan, Liechtenstein). In total, 5 tubes were placed for each hybrid ceramic sheet (n = 15). All samples were stored at 37°C for 24 hours in distilled water (Hygrobath, Whip Mix Louisville, KY, United States). Once the storage was completed, the mold tubes were carefully removed with #12 and #15 scalpel blades, exposing the resinous cement cylinders. All the samples were taken to a universal mechanical testing machine (Shimadzu AG-IS, Shimadzu Corporation, Kyoto, Japan), and by means of a steel wire handle, gauge 0.22 mm, with traction force, at a speed of 0.5 mm/min crosshead and a 50N load cell, the micro-shear test was performed. The bond strength was calculated using the equation: R = N/A, where R is bond strength given in MPa, N force in Newtons necessary for failure, and A is the area of the resin-cement-hybrid ceramic joint, measured in mm ^2^. The adhesive area (A) was determined by π.r2, where π is a constant and r is the radius obtained from the internal diameter of the medical-grade silicone tube.

All samples were evaluated in a stereomicroscope (NIKON SMZ800, Nikon Instruments Inc. New York, United States) at 40× and 50× to determine the type of failure, these were classified as: adhesive failure, i.e. where there is separation of the union between cement and ceramic, leaving an intact ceramic surface; cohesive failure in the ceramic, i.e where separation occurs only in ceramic; cohesive failure in the resin cement, i.e. where separation occurs only in cement; and mixed failure, which is a combination of the cohesive and adhesive patterns.

### Statistical analysis

For the statistical analysis, normality tests were carried out (Shapiro Wilk), and for the comparison of the groups, one-way ANOVA (Analysis of Variances) tests and Tukey's test were executed. A significance level of p < 0.05 was set. The R-project version R-3.6.3 for Windows was used for the statistical analyses (The R Project for Statistical Computing, St. Louis, Missouri, USA).

## Results

**Figure 1.**
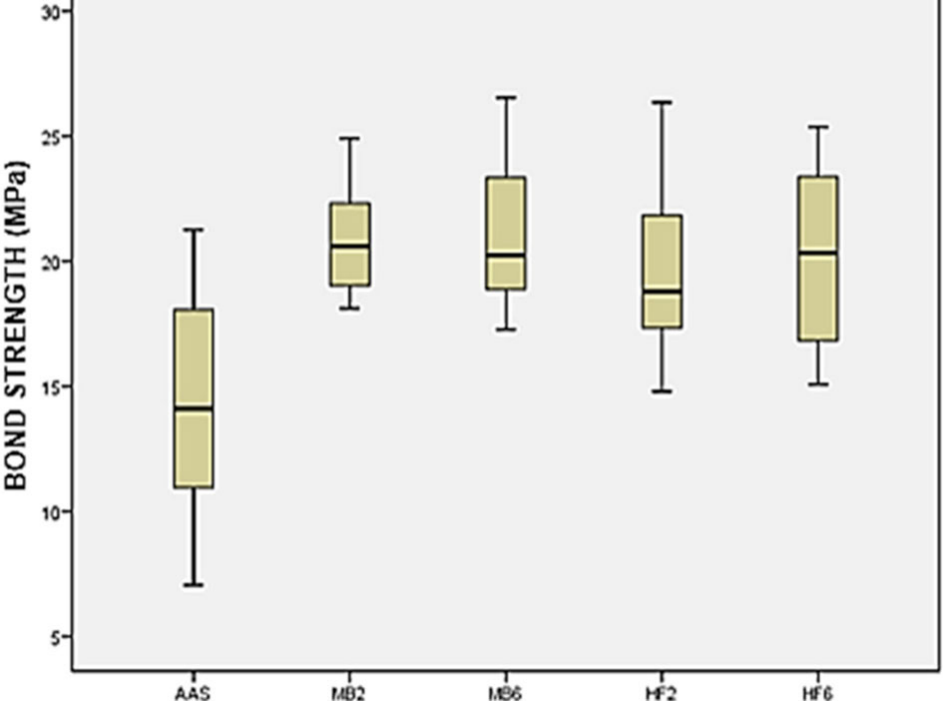
Box-plot bond strength (MPa). AAS: airborne-particle abrasion, HF2: hydrofluoric acid for 20s and silane, HF6: hydrofluoric acid for 60s and silane, MB2: a self-etch primer for 20s, MB6: a self-etch primer for 60s.

Descriptively, the results of the groups can be understood as follows: a graphical similarity in the mean values of bond strength the groups MB2, MB6, HF2 and HF6 was observed. the highest data dispersion was obtained in the AAS group, and the lowest graphic dispersion of data were obtained by both groups MB. The highest and lowest mean values of bond strength were reported by groups MB6 and AAS, respectively ( Figure 1).

**Figure 2.**
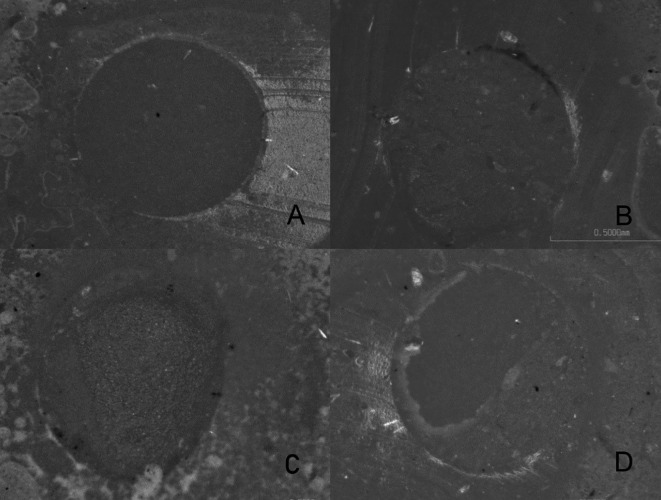
Example of each type of failure observed at the stereomicroscope (50×). A. Adhesive failure, B. Cohesive cement failure, C. Ceramic cohesive failure, D. Mixed failure.

In the statistical comparison by pairs (Tukey’s test) the groups that used Monobond Etch & Prime and hydrofluoric acid were considered equal, while the group AAS was statistically inferior compared to the other four groups ( Table 1). The predominant failure pattern in the study was mixed failure ( Table 2 and Figure 2). However, the AAS group exhibited an almost exclusive pattern of adhesive failure. For the other four groups, the mixed failure pattern was predominant. The full data are available from *Underlying data.* ^30^

**Table 1.** Micro-shear bond strength.

Groups	Mean (standard deviation)	95% confidence interval Lower limit	95% confidence interval Upper limit	Median	Variance	Min. value	Max. value	Range
AAS	14.41(4.33)A	12.01	16.81	14.09	18.81	7.04	21.24	14.20
MB2	20.73(2.03)B	19.60	21.86	20.58	4.14	18.10	24.89	6.79
MB6	21.11(2.70)B	19.61	22.61	20.23	7.32	17.27	26.54	9.26
HF2	19.66(3.47)B	17.73	21.58	18.78	12.10	14.79	26.34	11.55
HF6	20.21(3.66)B	18.18	22.24	20.32	13.44	15.07	25.36	10.28

AAS: airborne-particle abrasion, HF2: hydrofluoric acid for 20s and silane, HF6: hydrofluoric acid for 60s and silane, MB2: a self-etch primer for 20s, MB6: a self-etch primer for 60s. The groups with different letters in superscript (mean column) indicate statistically significant differences P<0.005. ANOVA F 10.124 gl 74 sig. 0.000.

**Table 2.** Failure type analysis (%).

Groups	n	Cohesive cement	Cohesive ceramic	Adhesive	Mixed
AAS	15	1 (6.6)	0 (0)	10 (66.6)	4 (26.6)
MB2	15	1 (6.6)	1 (6.6)	1 (6.6)	12 (80)
MB6	15	1 (6.6)	1 (6.6)	1 (6.6)	12 (80)
HF2	15	1 (6.6)	0 (0)	3 (20)	11 (73.3)
HF6	15	0 (0)	0 (0)	1 (6.6)	14 (93.3)

AAS: airborne-particle abrasion, HF2: hydrofluoric acid for 20s and silane, HF6: hydrofluoric acid for 60s and silane, MB2: a self-etch primer for 20s, MB6: a self-etch primer for 60s. The data are expressed in several samples that exhibited the type of failure and percentage in parentheses.

## Discussion

In the present study, the bond strength between a PICN and a resinous cement after the use of hydrofluoric acid, a self-etching ceramic primer, and airborne-particle abrasion was compared. The airborne-particle abrasion group was significantly inferior to the two groups of acid etching agents. Therefore, the hypothesis that raised the statistical equality of the mean values of bond strength in the three surface treatments was not accepted.

Airborne-particle abrasion with aluminum oxide particles is an alternative to increase the roughness of the ceramic substrate by impulsing and impacting the surface with abrasive particles, with the potential risk of microcracks and excessive damage to the treated surface. ^13^ Parameters such as distance, time, pressure, and particle size can result in different roughness patterns. ^13^^,^^14^ Abrasion with 30 μm size aluminum oxide has been associated with high roughness values and acceptable bond strength mean values on PICN. ^15^ Although in the literature it is also reported that in CAD-CAM materials with polymeric matrix and dispersed ceramic particles, airborne-particle abrasion with aluminum oxide has shown superior results to acid etching. In PICN an opposite phenomenon generally occurs, this is perhaps based on the microstructural organization of this material. ^16^ The use of airborne-particle abrasion on the surface of a hybrid ceramic creates roughness through impact microcracks, unlike acid etching that creates micropores due to the partial dissolution of the vitreous matrix. ^17^ Etching with hydrofluoric acid and airborne-particle abrasion with aluminum oxide, followed by silane, has been reported with surface energy values in Enamic of 147 mJ/m ^2^ and 96 mJ/m ^2^ respectively. This suggests that the amount of roughness in a surface is not predictive of higher bond strength. ^18^ Another possible explanation is that VITA ENAMIC, unlike other hybrid CAD-CAM materials, contains a porous network of feldspathic ceramic reinforced with alumina and only one infiltrate of urethane dimethacrylate polymer, allowing a better action of the etching agent and assuming the increased risk of surface damage and unevenness if airborne-particle abrasion is used. ^19^ This mechanism probably explains why, even though both methods can create comparable values of surface roughness in a hybrid ceramic, the bond strength results can be higher after a chemical attack by acid. ^9^^,^^20^ Only one protocol of air abrasion with aluminum oxide was contemplated in this study, which could be considered as a limitation of the research.

On the other hand, the self-etching ceramic primer in the literature has reported bond strength results comparable to hydrofluoric acid and silane on polymer infiltrated ceramic network. ^20^^,^^21^ Both etching agents can produce similar roughness values, but self-etching ceramic primer was introduced to the market as a less aggressive alternative to hydrofluoric acid. ^12^^,^^22^ The physical-chemical action is due to an acid etching with tetrabutylammonium dihydrogen trifluoride and a chemical interaction through trimethoxy propyl methacrylate, without diminishing the mechanical resistance and fatigue of the ceramic substrate. ^23^ Additionally, a formation of a hydrophobic silane layer that is theoretically more resistant to hydrolytic degradation has been associated with the use of this self-etching ceramic primer. ^24^ The etching depth of 9% hydrofluoric acid for 20 and 60s is greater than 290 μm compared to a depth of 7 μm of Monobond Etch & Prime for 40s in VITA ENAMIC. ^25^ Therefore, the indication of which surface treatment to use does not only depend on the bond strength results obtained. Hydrofluoric acid on VITA ENAMIC is dependent on the time and concentration of use. Hydrofluoric acid concentrations of 5% between 30 and 90 seconds do not seem to significantly affect flexural strength, on the contrary, the use of hydrofluoric acid at 10% after 30 seconds reports a significant decrease in flexural strength of this hybrid ceramic. ^26^ In this study a concentration of 9.6% was used, with similar bond strength results between 20 and 60 seconds. A consistent finding that hydrofluoric acid etching of VITA ENAMIC for more than 30 seconds does not significantly improve bond strength by shear test. ^5^ This analysis may be somewhat helpful in supporting the recommendation of the lowest possible time and concentration of hydrofluoric acid to treat the surface of VITA ENAMIC. However, the present investigation had the limitation of only evaluating one hydrofluoric acid concentration.

Adhesive failure patterns have been associated with low values of bond strength, cohesive and mixed patterns seem to be considered more acceptable because they suppose a better infiltration of the resinous cementing agent in the conditioned surface of the PICN. ^27^^,^^28^ In the present investigation, the predominant pattern was mixed for the hydrofluoric acid and self-etching ceramic primer groups, consistent with the highest values of bond strength in the study, and an almost exclusive pattern of adhesive failures for the airborne-particle abrasion group, in which the lowest values were obtained. The results of this investigation should be considered with caution since the bond strength test was carried out after the short-term storage of the samples. Bond strength can be affected by hydrolytic degradation of the materials in the small adhesive area, storage in water for long periods of time, or thermal cycling. ^28^^,^^29^

## Conclusions

With the limitations of the present investigation, the self-etching ceramic primer and hydrofluoric acid followed by silane were reported to be statistically equal at 20 and 60 seconds. The 20 second time seems to be a more reasonable option than 60 seconds applying these surface treatments on a PICN. This is because, if the same bond strength result is obtained at 20 and 60 seconds, it can increase the surface energy in less time. Airborne-particle abrasion is not recommended as the first choice of surface treatment in a PICN.

## Data availability

### Underlying data

Mendeley Data: Micro-shear bond strength of different surface treatments on a polymer infiltrated ceramic network. https://doi.org/10.17632/dfm9wr4bwn.4. ^30^

This project contains the following underlying data:

– Acronym manuscript Micro-shear bond strength of different surface treatments on a polymer infiltrated ceramic network.docx (description of acronyms used within the data spreadsheets).
– Statistical Data.xlsx (raw data for each group. AAS is airborne-particle abrasion, MB2 is a self-etch primer for 20 seconds, HF2 is 9.6% hydrofluoric acid for 20 seconds, MB6 is a self-etch primer for 60 seconds, HF6 is 9.6% hydrofluoric acid for 60 seconds).
– Description groups.xlsx (statistical examination as a comparison among the groups. These statistical examinations are ANOVA, post hoc ANOVA using Tukey, and the graph of the groups).
– Comparison adhesive resistance groups.xlsx (statistical descriptive among the groups. Shows the components in the statistical descriptive, such as median, kurtosis, percentiles, etc. that show the explore descriptive data among these group).

Data are available under the terms of the Creative Commons Attribution 4.0 International license (CC-BY 4.0).
