# Supplementary material for: Micro-shear bond strength of different surface treatments on a polymer infiltrated ceramic network
Source: F1000Res. 2022 Jul 18;11:798. [Version 1] doi: 10.12688/f1000research.122108.1 (PMC10665602; doi:10.12688/f1000research.122108.1)
Supplement: Peer review history

# Reviewer report: Reviewer response for version 1

**DOI:** 10.5256/f1000research.134054.r215076

**Published:** 2023-11-22

**Authors:** Sivaranjani Gali

**Affiliations:** 1 M.S. Ramaiah University of Applied Sciences, Bengaluru, Karnataka, India

The authors investigated the micro-shear bond strength of different surface treatments on a polymer infiltrated ceramic network, especially of VITA Enamic. 

 The following are my comments:

1. Schematic illustrations are required to differentiate the groups
2. Conclusion can be rephrased in line with the objective of the study. 
3. Flowchart of methodology to be included. 
4. Were the samples thermocycled ?
5. Discussion can include the results of similar studies and how this study stands out from the rest?
6. Mechanism of action of sandblasting and acid etching can be illustrated. 

Is the work clearly and accurately presented and does it cite the current literature?

Partly

If applicable, is the statistical analysis and its interpretation appropriate?

I cannot comment. A qualified statistician is required.

Are all the source data underlying the results available to ensure full reproducibility?

Yes

Is the study design appropriate and is the work technically sound?

Partly

Are the conclusions drawn adequately supported by the results?

Partly

Are sufficient details of methods and analysis provided to allow replication by others?

Partly

Reviewer Expertise:

Dental ceramics

I confirm that I have read this submission and believe that I have an appropriate level of expertise to confirm that it is of an acceptable scientific standard, however I have significant reservations, as outlined above.

# Reviewer report: Reviewer response for version 1

**DOI:** 10.5256/f1000research.134054.r215064

**Published:** 2023-11-20

**Authors:** Guillermo Manuel Herrera Pérez

**Affiliations:** 1 Centro de Investigación en Materiales Avanzados, CONAHCYT, Chihuahua, Mexico

I read the manuscript entitled “Micro-shear bond strength of different surface treatments on a polymer infiltrated ceramic network” written by Wahjuningrum *et al*. The authors show the interesting results. They evaluate the effect of hydrofluoric acid and a self-etching ceramic primer, considering two different application times. Also, they evaluate the influence of both surface conditions on the bond strength of a polymer-infiltrated ceramic network. The manuscript is well organized, and the references used are fine in number and actual. The authors describe the preparation of samples, and they describe the methodology to determine the bond strength. The current work is complemented by the determination of the type of failure (adhesive failure, cohesive failure, and mixed failure) considering the stereomicroscope technique. The predominant failure pattern was a mixed failure. In general, the statistical analyses and their interpretation seem to have been performed properly and the conclusions reached are in line with the results presented. The methods and analyses provided in this manuscript can be replicated by others. Certainly, the theme of this manuscript is suitable for the journal and surely of wide interest to the reading community of F1000Research.

 The paper is acceptable after minor revisions for indexing after the comments below are addressed.

1. The scale bar in the stereomicroscope images should be clearer (Figure 2). 
2. Atomic force microscopy (AFM) analysis should be implemented. The authors mention, in the discussion section, "different roughness patterns, high roughness, and surface roughness", which needs to be verified (including micrographs) and quantified. Additionally, AFM analysis can provide information about physical defects such as microcracks. Also, this technique allows us to evaluate the presence of micropores.
3. The authors focus on the effect of airborne particle abrasion on the surface of a hybrid ceramic. However, the average particle size (including micrographs and histogram analysis) influence on roughness is not investigated. Please conduct transmission electron microscopy analysis.

Is the work clearly and accurately presented and does it cite the current literature?

Yes

If applicable, is the statistical analysis and its interpretation appropriate?

Yes

Are all the source data underlying the results available to ensure full reproducibility?

Yes

Is the study design appropriate and is the work technically sound?

Yes

Are the conclusions drawn adequately supported by the results?

Partly

Are sufficient details of methods and analysis provided to allow replication by others?

No

Reviewer Expertise:

Ceramics (ferroelectric and ferromagnetic), Raman, and EELS spectroscopies.

I confirm that I have read this submission and believe that I have an appropriate level of expertise to confirm that it is of an acceptable scientific standard, however I have significant reservations, as outlined above.

# Reviewer report: Reviewer response for version 1

**DOI:** 10.5256/f1000research.134054.r210203

**Published:** 2023-11-20

**Authors:** Nantawan Krajangta

**Affiliations:** 1 Thammasat University, Pathum Thani, Thailand

This study examines the micro-shear bond strength of various PICN surface treatments. This is an *In Vitro* study, which has potential implications for clinical applications in restorative dentistry using the PICN. The clarity and organization of the manuscript are commendable. I have only minor suggestions for improvement as follows:

 Abstract:

1. To improve the coherence of the abstract, it is recommended to align the sequence of description with the order of experimentation and results. Specifically, the abstract currently discusses HF2 and HF6 before MB2 and MB6, while the presentation of experimental details and results showcases MB2 and MB6 before HF2 and HF6. To enhance consistency, consider revising the abstract to ensure that the order of describing treatments aligns with the order in which the experimentation and results are presented.

Introduction:

1. The introduction section is well structured. However, to improve this section, explain and emphasize existing knowledge that has been studied and the gap of knowledge related to surface treatment of PICN.

Methodology:

 1. Research design:

1. Please explain more about how the manual pressure is uniformly controlled for polishing the specimens.
2. It is typically preferred to prepare samples with width and length matching the block dimensions, but the sample in this study has dimensions of 8x10x2 mm. It was prepared by cutting the VITA ENAMIC EM14 block with dimensions of 12x14x18 mm. Please explain the cutting method. To prepare additional samples in the content or use figures.

2. Experimental procedure:

1. The introduction states, "Etching with 5% hydrofluoric acid for 60 seconds is the PICN manufacturer's recommended surface conditioning." However, the testing involved the use of 9.6% HF. Could you provide the rationale for this choice?
2. In this study, all samples underwent evaluation using a stereomicroscope (NIKON SMZ800, Nikon Instruments Inc. New York, United States) at both 40x and 50x magnifications to ascertain the type of failure. Why was testing conducted at both 40x and 50x, especially considering that study results are only presented at 50x magnification?

   

 Result:

1. The order of abbreviation descriptions in Tables 1 and 2 should be modified to reflect the order of information presented in the tables.

Discussion:

1. This study on the effects of 9.6% hydrofluoric acid, therefore, the statement "This analysis may be somewhat helpful in supporting the recommendation of the lowest possible time and concentration of hydrofluoric acid to treat the surface of the VITA ENAMIC." is inaccurate and does not align with the outcomes of this study. Please correct this statement.

Is the work clearly and accurately presented and does it cite the current literature?

Yes

If applicable, is the statistical analysis and its interpretation appropriate?

Yes

Are all the source data underlying the results available to ensure full reproducibility?

Yes

Is the study design appropriate and is the work technically sound?

Partly

Are the conclusions drawn adequately supported by the results?

Partly

Are sufficient details of methods and analysis provided to allow replication by others?

Partly

Reviewer Expertise:

digital dentistry, esthetic dentistry, dental materials

I confirm that I have read this submission and believe that I have an appropriate level of expertise to confirm that it is of an acceptable scientific standard.
